# Female athletes explicitly gesture in emotional situations

**DOI:** 10.3389/fpsyg.2024.1526542

**Published:** 2025-01-07

**Authors:** Y. Adams, M. Augenstein, P. Furley, A. Krieg, P. Born, I. Helmich

**Affiliations:** ^1^Department of Motor Behavior in Sports, Institute of Health Promotion and Clinical Movement Science, German Sport University Cologne, Cologne, Germany; ^2^Institute of Training and Computer Science in Sport, German Sport University Cologne, Cologne, Germany; ^3^Institute of Professional Sport Education and Sport Qualifications, German Sport University Cologne, Cologne, Germany; ^4^Department of Exercise and Sport Studies, Smith College, Northampton, MA, United States

**Keywords:** nonverbal movement behavior, emotions, gestures, sports, gender

## Abstract

**Introduction:**

Both appraisal emotion approaches and self-regulation theory emphasize that appraising an event as conducive or detrimental to one’s current goals may trigger an affective response that can be observed nonverbally. Because there may be a female advantage in the inhibition and self-regulation of emotions, we hypothesized that female but not male athletes regulate emotions during sports through explicit nonverbal behaviors.

**Methods:**

All nonverbal hand movement behavior of right-handed female and male tennis athletes was recorded during competitive matches. All immediate nonverbal expressions after point losses and wins were coded by two independent blind raters applying the NEUROpsychological GESture (NEUROGES®) system.

**Results:**

No gender differences were found for overall hand movement activity. Female athletes executed more fall gestures than males as well as in space and both-handed act as a unit hand movements. In contrast to males, female athletes spent significantly more time with both-handed pantomime gestures (e.g., performing an imaginary backhand), particularly when losing points.

**Discussion:**

Increased expressions of pantomime gestures in female athletes after losing indicate that women regulate negative emotions nonverbally through explicit hand movements. Thus, female athletes seem to nonverbally cope with their negative emotional arousal through explicit nonverbal behaviors in order to control performance.

## 1 Introduction

Sport competitions are replete with emotional instances and emotional expressions, which is likely one of the reasons why millions of spectators sit spellbound in front of the television or flock to large sports arenas and live public viewing events. Sports, therefore, provide an opportune context to enhance understanding of the emotions and nonverbal behavior of humans in a real-life context (Furley, 2018). This reasoning is supported by numerous studies that used the sport context as a means to advance theoretical understanding of emotions and their nonverbal expressions (Aviezer et al., 2012; Drewes et al., 2020; Matsumoto and Willingham, 2006; Neumann et al., 2021). The present study attempts to build on this initial research to gain insight into specific observable behaviors associated with different emotional states in tennis and, additionally, test for gender differences in emotional expressions.

Pertinent to the present research, studies have shown that experiencing an emotion often shows in a person’s observable behavior (Darwin, 2009; Ekman, 1992). In this case, scholars typically describe the observable behavior as nonverbal behavior, which is loosely defined as all expressive movements including facial, vocal, and postural expressions, as well as touch, proxemics, and gaze (Darwin, 2009; Ekman, 1977; Lausberg, 2013, 2019). It is important to note that nonverbal behavior can convey many other kinds of information, such as information relevant to opinions, values, personality dispositions, psychopathologies, physical states such as fatigue, and cognitive states such as comprehension or interest (Densing et al., 2018; Fridlund, 1994; Helmich et al., 2014, 2021, 2024; Helmich et al., 2020a; Helmich et al., 2020b; Helmich and Lausberg, 2019; Helmich and Schepmann, 2023; Hogrefe et al., 2016; Lausberg and Kryger, 2011; Neumann et al., 2017). Hence, the dominant theoretical notion that nonverbal behavior - particularly facial expressions, but also vocal, postural expressions, touch, proxemics, and gaze—directly expresses emotions has come under increasing scrutiny (Fridlund, 1994; Russell et al., 2003). Without going into unnecessary detail of this central debate in the field of emotions, there is solid evidence that certain circumstances (e.g., winning and losing a point or a competition in sport) lead to different emotional experiences and different nonverbal expressions (Aviezer et al., 2012; Drewes et al., 2020; Matsumoto and Hwang, 2012; Matsumoto and Willingham, 2006; Moesch et al., 2015; Moll et al., 2010; Neumann et al., 2021; Tracy and Matsumoto, 2008). Hence, it seems reasonable to conclude that an athlete’s post-performance nonverbal behavior is closely related to their affective state (Fritsch et al., 2022). This reasoning is in line with appraisal emotion approaches (Lazarus, 1991; Scherer, 2013) as well as self-regulation theory (Carver and Scheier, 1990), which emphasize that appraising an event as conducive or detrimental to one’s well-being and/or current goals may trigger an affective response. Applied to the present research, such theorizing suggests that winning a point in tennis would likely be appraised as conducive to the aim of winning a competition and, in turn, trigger a positive affective response. In contrast, losing a point would likely be appraised as detrimental to this aim and, thus, trigger a negative affective response. Hence, the present research investigated the nonverbal behaviors of male and female tennis players after winning or losing a point in tennis.

Since Darwin’s (1872/2009) book on the expression of emotions (Darwin, 2009), many scholars have been interested in the spontaneous and automatic expressions of emotional states. However, when considering nonverbal post-performance expressions in tennis, it is important to note that nonverbal behavior is expressed either implicitly or explicitly (Drewes et al., 2020; Neumann et al., 2021). In fact, there is general consensus that nonverbal behavior is under both conscious (/explicit), deliberate control, and unconscious (/implicit), autonomous control [e.g., see Lausberg (2013) and Matsumoto et al. (2013) for a review], which can be differentiated between explicit and implicit nonverbal hand movements and gestures (Helmich et al., 2014, 2024; Helmich et al., 2020a; Helmich et al., 2020b; Helmich and Lausberg, 2019; Helmich and Schepmann, 2023; Lausberg, 2013).

### 1.1 The present research

Most emotion expression studies have focused on facial expressions (Matsumoto et al., 2013) given the availability and popularity of the Facial Action Coding System (FACS: Ekman and Friesen 1967, 1982) and the methodological difficulties in the field of body movement research due to the large degrees of freedom in whole-body movements. Although the face can certainly be regarded as an important channel for communicating affective states in humans, recent research has indicated that the body might be a more reliable source in informing observers about intense emotional moments in sports (e.g., Aviezer et al., 2012).

A series of studies has investigated the nonverbal behaviors of Olympic or Paralympic Judoka after winning or losing fights (Matsumoto, 2009; Matsumoto and Willingham, 2006; Tracy and Matsumoto, 2008). These studies provided evidence for a specific pattern of body movements in the face and body that both blind and sighted athletes show after winning and losing entire fights. However, these (potentially universal) expressions directly after competition were shown to be deliberately modified according to display rules (e.g., during ceremonies) and differed depending on the culture of the athletes (Matsumoto, 2009). Together these studies suggest that humans seem to be biologically prepared in advance of experience to encode certain affective information in their bodily movements after winning or losing important fights. However, this encoding of affective information in athletes’ bodily action can be modified by learning and cultural experience. Further related research has shown that different contexts in sport result in different nonverbal expressions, for example when scoring goals in handball (Moesch et al., 2015), after penalties in football (Moll et al., 2010), and/or when winning and/or losing points in tennis (Aviezer et al., 2012; Drewes et al., 2020; Neumann et al., 2021).

Of particular importance to the present research, Neumann et al. (2021) reported first evidence that professional tennis players show a particular lateralized nonverbal behavior with the right hand as a response to positive affect after winning a point in tennis. In addition, the study found that point losses were accompanied by particular nonverbal movement behaviors such as irregular on-body hand movements, mostly executed with the left hand. The data of Neumann et al. (2021) suggested that winning and losing in professional tennis is not only characterized by particular nonverbal expressions but that nonverbal hand movements and gestures of athletes serve different neuropsychological functions, i.e., winning points leads to positive affective states that are nonverbally expressed by body-distant gestures but change toward their own body to regulate stress when losing (Neumann et al., 2021). Hence, the first aim of the present research was to scrutinize this theory and test the hypothesis that positive affective states (after winning points) leads to lateralized body-distant movements and that negative affective states (after losing a point) leads to on-body movements in an attempt to regulate these negative affective states in semi-professional athletes.

The second theoretical aim of the present research was to test for gender differences in affective nonverbal behavior in tennis. Stereotypical beliefs that women are more emotional than men have been reported in literature (Belk and Snell, 1986; Birnbaum et al., 1980; Heesacker et al., 1999). This belief has been suggested to particularly show in behavioral expressions of emotions (Briton and Hall, 1995). However, gender differences in emotional expressions might not be the same for all emotions and might differ depending on emotional valence. In this respect, it is, for example, believed that women smile more and express more warmth and affection than men (Briton and Hall, 1995). On the other hand, men were believed to be louder and more interruptive and to display more nervous, dysfluent behaviors (Briton and Hall, 1995). It is also believed that women express more fear, vulnerability, and sadness than men, whereas men are expected to be more aggressive and express more anger than women (Briton and Hall, 1995; Fabes and Martin, 1991). However, real-world investigations of the nonverbal behavior between genders are very rare and there are hardly any descriptive coding studies that have scrutinized differences in bodily movements in real-world affective situations. Thus, the intention of this study is to investigate if nonverbal emotional expressions underlie gender effects and if the hands serve different neuropsychological functions during the experience of positive or negative emotional states in female and male athletes.

Although there is reason to belief that men and women express affective states differently, the present research investigates these gender difference in an explorative manner. According to Bjorklund and Kipp's (1996) hypothesis, females may have evolved a greater ability to inhibit prepotent responses. In fact, a female advantage exists in behavioral as well as social inhibition such as, for example, during the control of emotions (Bjorklund and Kipp, 1996; Hosseini-Kamkar & Bruce Morton, 2014). Nonverbal hand movements and gestures can serve to self-regulate during stress and emotions (Barroso et al., 1978; Densing et al., 2018; Helmich and Lausberg, 2019; Helmich and Schepmann, 2023; Neumann et al., 2021; Reinecke et al., 2020). Thus, when compared to men, woman may gesture more in order to nonverbally control / self-regulate emotional situations. To test this hypothesis during real life scenarios, we investigated female and male athletes during emotional situations such as when winning or losing points during competitive tennis matches. We hypothesized that women express more explicit hand movements and gestures when compared to male athletes in order to nonverbally control performance during emotional situations in sports.

## 2 Methods

### 2.1 Sample

An *a priori* power analysis (with G*Power 3.1.9.7) indicated that 20 participants are necessary for a statistical analysis between groups and repeated measures (effect size *f* = 0.35, calculated critical *F* value = 2.775, calculated actual Power = 0.96). Thus, 20 semi-professional tennis players [N (female) = 10; N (male) = 10] were videotaped during matches of the 2020 German “Oberliga” (4th German league) season. Athletes were all right-handed and, on average, 26.75 (± 6.96) years old. All participants signed an informed consent form for video and audio recording during the study. The study was approved by the local ethics committee (Nr. 125 / 2019).

### 2.2 Video recordings

**Figure 1:**
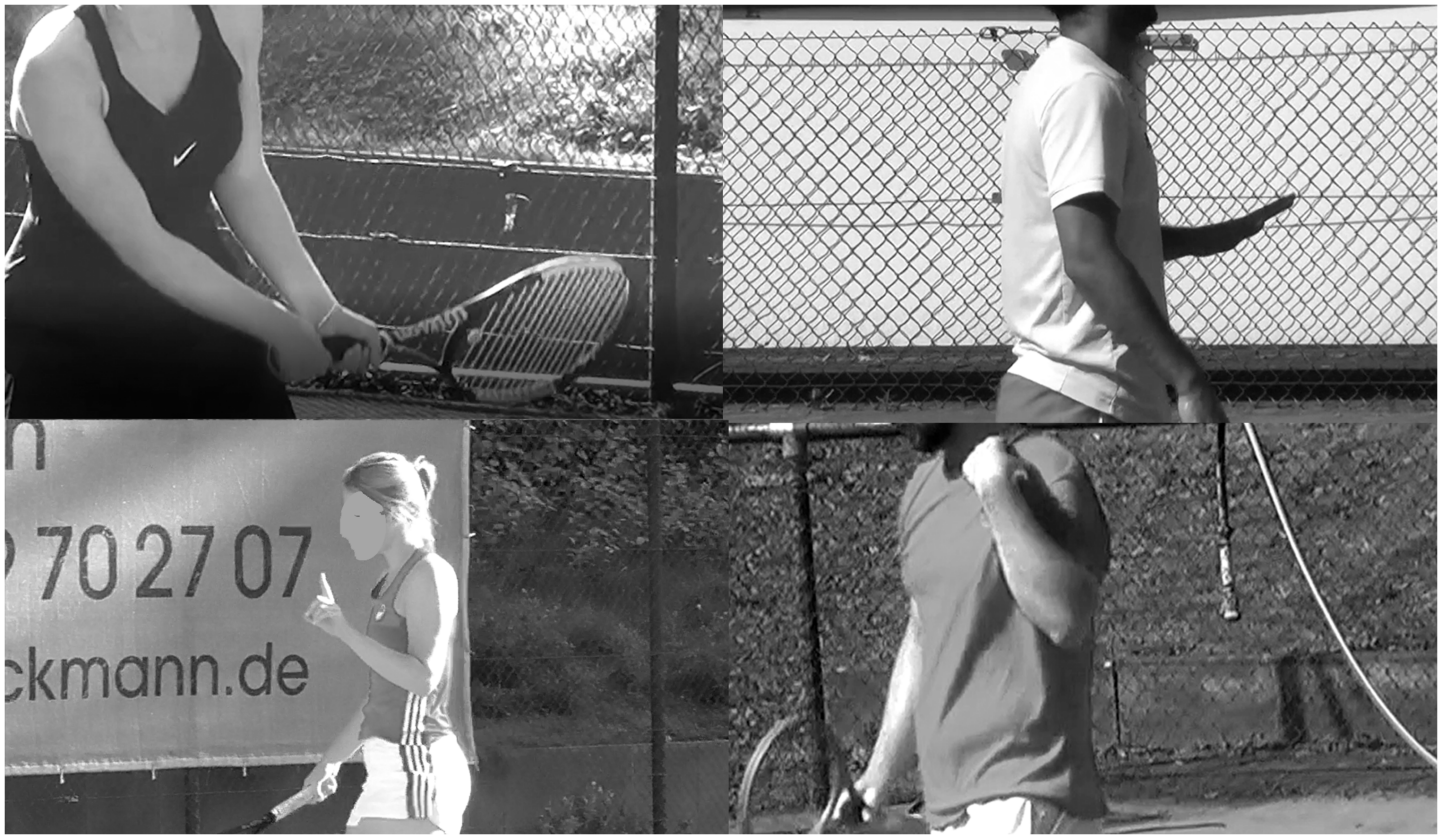
Exemplary nonverbal behaviors of athletes during real match situations in tennis.

We recorded tennis athletes between points during regular league tennis matches on outdoor tennis courts (Figure 1). Tennis offers the unique situation of a relatively controlled situation of 25 s between point games (International Tennis Federation, 2023). After each point game, an athlete will have just won or lost a point. These two situations in tennis are associated with positive (winning a point) and negative (losing a point) emotional experiences (Aviezer et al., 2012; Jekauc et al., 2021; Lewis et al., 2017) and nonverbal expressions (Drewes et al., 2020; Neumann et al., 2021). Thus, we used winning and losing a point in tennis as the positive or negative emotional experiences of each athlete. All video recordings were checked and sorted out with respect to the following exclusion criteria: blurred or shaky footage, player had to react to circumstances that were not related to the match (e.g., tying shoelaces), the result of the played point was in doubt and needed to be proofed by the chair umpire, the video did not show the whole player, or the video duration was too short. We then randomly selected 20 post-point behaviors (each 4 s long) of each athlete: 10 after losing a point and 10 after winning a point. The coding time of athletes’ behavior was defined to 4 s post-point because spontaneous emotional expressions last between 0.5 and 4 s (Ekman and Friesen, 1982; Frank and Ekman, 1993; Matsumoto and Willingham, 2006; Richardson et al., 2000).

### 2.3 Measures and coding procedure

The videos were coded for nonverbal hand movement and gestural behavior using the NEUROpsychological GESture (NEUROGES®) system (Lausberg, 2013, 2019). Previous data show that the analysis system is objective and reliable in clinical and cognitive research as well as in the sports setting (Drewes et al., 2020; Helmich et al., 2020a, 2021, 2022; Helmich et al., 2023; Helmich and Lausberg, 2019; Neumann et al., 2021). Two independent, certified, and blinded raters coded the videos without sound and without outcome reference across all three modules of the NEUROGES. Rater 1 (R1) coded 100% of the videos. R2 coded 20% of the videos to calculate inter-rater agreement (IA) between R1 and R2. The IA was calculated with the modified Cohen’s kappa according to Holle and Rein (2015). This modified Cohen’s kappa takes into account not only the categorization of values but also the temporal overlap of the raters’ annotations (Holle and Rein, 2015). Results of the IA are presented as the modified Cohen’s kappa and the raw agreement is shown in Table 1. The raw agreement represents the number of agreeing cases divided by the total number of cases. The agreement in the present investigation was most of the time “substantial” (“0.61–0.80″) and “almost perfect” (“0.81–1.00″; in terms of Landis and Koch (1977)) and referenced previous scores (Drewes et al., 2020; Helmich et al., 2020a, 2021, 2022; Helmich and Lausberg, 2019; Neumann et al., 2021). The analysis of nonverbal hand movements and gestures concerned all three modules of the NEUROGES analysis tool (Lausberg, 2013; Table 1). Due to the specificity of the sports setting and the shortness of the video, the individual arm swing and movements that had only a preparation or retraction phase caused by the video editing were not coded.

**Table 1:** Short definitions of the NEUROGES categories according to Lausberg (2013, 2019) with the Inter-rater agreement (IA) for each value (according to Holle and Rein, 2015).

Category	Short definition	IA (/raw agreement) for the right (RH), left (LH), and both hands (BH)
Activation		
Movement	hands in active motion	RH: 0.66 LH: 0.70
Structure		
Irregular	small movements without distinct trajectory, potentially ongoing time	RH: 0.56 / 0.98 LH: 0.62 / 0.97
Repetitive	movement with a phase structure and a repetitive motion complex phase	RH: 0.91 / 0.98 LH: 0.79 / 0.98
Phasic	movement with a phase structure and a static or phasic motion complex phase	RH: 0.61 / 0.88 LH: 0.66 / 0.89
Aborted	disrupted transport phase or shift followed by retraction	RH: no units LH: 1.00 / 1.00
Focus		
Within body	acting on body-internal structures	RH: no units LH: 0.00 / 0.98
On body	acting on the body surface	RH: 0.81 / 0.98 LH: 0.65 / 0.88
On attached object	acting on an object that is attached to the body	RH: 0.80 / 0.99 LH: 0.69 / 0.92
On separate object	acting on an object that is separate from the body	RH: 0.56 / 0.78 LH: 0.69 / 0.91
In space	acting in space without touching something	RH: 0.59 / 0.84 LH: 0.56 / 0.83
Contact		
Act as a unit	both hands are in touch with a fixed configuration and take a joint action	BH: 0.93 / 0.99
Act apart	both hands act simultaneously without touching each other	BH: 0.67 / 0.93
Formal relation		
Symmetrical	both hands move on symmetrical trajectories	BH: 0.94 / 0.99
Right hand dominance	the right hand is dominant	BH: 0.80 / 0.95
Left hand dominance	the left hand is dominant	BH: 0.73 / 0.93
Asymmetrical	Both hands move on asymmetrical trajectories and are equally dominant	BH: 0.75 / 0.88
Function		
Emotion/attitude	displaying exclusively an emotion or an attitude	BH: 0.88 / 0.99 RH: 0.75 / 0.97 LH: 0.82 / 0.98
Emphasis	setting accents on speech	BH: no units RH: no units LH: 1.00 / 1.00
Egocentric direction	indicating a direction or route by using an egocentric frame of reference	BH: 1.00 / 1.00 RH: 1.00 / 1.00 LH: 1.00 / 1.00
Pantomime	pretending to perform an action	BH: 1.00 / 1.00 RH: 1.00 / 1.00 LH: 1.00 / 1.00
Object-oriented action	changing the external physical world	BH: 0.92 / 0.96 RH: 0.72 / 0.89 LH: 0.90 / 0.95
Subject-oriented action	Changing the own physical (and secondarily mental) state	BH: 0.56 / 0.98 RH: 1.00 / 1.00 LH: 0.89 / 0.95
Emblem / social convention	Using culture-specific hand signs with conventionalized arbitrary meanings / conventionalized actions in specific social contexts	BH: 1.00 / 1.00 RH: 1.00 / 1.00 LH: 1.00 / 1.00
Type		
Rise	Dynamic fast raising up of the arms (against gravity)	BH: no units RH: 0.80 / 0.99 LH: 1.00 / 1.00
Fall	Letting the arms fall down heavily (giving in to gravity)	BH: 0.90 / 0.99 RH: 0.86 / 0.98 LH: 0.82 / 0.98
Clap/beat	Dynamic fast strong movement of the arms	BH: no units RH: 1.00 / 1.00 LH: no units
Baton	Small up down movements with upward accent	BH: no units RH: no units LH: 0.00 / 0.99
Palm-out	Small supination-pronation movements with outwards accent	BH: no units RH: no units LH: 1.00 / 1.00
Neutral	Indicating a direction without specifying an agent	BH: 1.00 / 1.00 RH: 1.00 / 1.00 LH: 1.00 / 1.00
Transitive	Acting as if with an imaginary (or real) object or counterpart	BH: 1.00 / 1.00 RH: 1.00 / 1.00 LH: 1.00 / 1.00

Module I of the NEUROGES consists of the three steps: Activation, Structure, and Focus. Activation describes muscular activation in motion using the values movement and no movement of the right and left hands. It measures the extent of a person’s psychomotor activity (Lausberg, 2013, 2019). The Structure category classifies movement values based on trajectory and dynamics into five subcategories: phasic, repetitive, shift, aborted, and irregular. The presence of movement phases (preparation, complex, retraction) provide information about different levels of cognitive complexity (Lausberg, 2013, 2019). Whereas phasic and repetitive hand movements are characterized by the three movement phases, shift, irregular, and aborted hand movements do not contain three movement phases. In the Focus category, the three Structure values irregular, repetitive, and phasic are defined based on the locality of their complex phase with six subcategories: within body, on body, on attached object, on separate object, on person, and in space.

Module II consists of the two categories Contact and Formal Relation. The use of the hands with and in relation to each other allows conclusions to be made regarding the laterality and interhemispheric coordination of movement concepts (Lausberg, 2013, 2019). Contact describes the physical contact of the hands, which gives an indication of the level of bihemispheric sensorimotor activation of the values: act on each other, acta as a unit, and act apart (Lausberg, 2013, 2019). Formal Relation provides the basis for the assessment of cognitive concepts with the description of the dominance (values: right hand dominance, left hand dominance, symmetrical, asymmetrical) of the hands. For the analysis of Formal Relation, only phasic and repetitive hand movements are evaluated.

Module III consists of the two steps Function and Type. It provides an analysis of conceptual body movements. Body movements do not happen randomly; they show interaction with emotions, cognitions, and interactive processes (Lausberg, 2013, 2019). Function describes gestures and actions with the following values: emotion/attitude, emphasis, egocentric deictic, egocentric direction, pantomime, form presentation, spatial relation presentation, motion quality presentation, object-oriented action, subject-oriented action, and emblem/social convention. To further differentiate, 24 Type values were given up to four subcategories for each Function value (e.g., emotion/attitude—fall; pantomime—transitive; Table 1).

### 2.4 Statistics

The data was exported and analyzed according to the guidelines of the NEUROGES-Elan system (Sassenberg and Helmich, 2013). Each NEUROGES category (e.g., Structure with its single value such as, for example, phasic) was statistically analyzed by repeated measures analysis of variance (rmANOVA) and univariate analysis of variance (uANOVA) using SPSS (IBM SPSS Statistics version 25) with the within-subjects factors hand [right (rh) vs. left hand; lh; also both hands (bh) for module II and module III] and emotion (winning vs. losing a point) and the between-subjects factor group (female vs. male). The statistical analysis of the nonverbal hand movement and gesture values was performed using frequency (F) and proportion of time (PoT). The frequency of value units per video was calculated by the mean value unit frequencies of each player divided by the duration of the videos (units/video). The proportion of time of value units per video minute was calculated by the mean value unit duration in seconds of each player divided by the video duration in minutes (seconds/min). *Post-hoc* pairwise comparisons were Bonferroni-corrected. If a value occurred fewer than five times it was not included in the statistical analysis. Thus, the following values were included in the statistical analysis: movement (Activation); phasic, repetitive, irregular, aborted (Structure); in space, on attached object, on separate object, on body (Focus); act as a unit, act apart (Contact); symmetrical, asymmetrical, right hand dominance, left hand dominance (Formal Relation); emotion/attitude, emphasis, egocentric direction, pantomime, object-oriented action, subject-oriented action und emblem/social convention, and (Function); rise, fall, calp/beat, baton, palm-out, neutral, and transitive (Table 1).

## 3 Results

### 3.1 Research question 1

*How do nonverbal behaviors differ in semi-professional athletes during positive and negative affect situations?*

**Activation**. The rmANOVA for the frequency values showed a significant effect of the hand [*F*(1, 18) = 7.607, *p* < 0.05, η^2^ = 0.297; Table 2]. *Post-hoc* pairwise comparisons showed that athletes executed significantly more right-handed movements when compared to executions with the left hand (*p* < 0.05). The rmANOVA for the PoT values showed a marginal effect of the hand [*F*(1, 18) = 3.877, *p* = 0.065, η^2^ = 0.177]. *Post-hoc* pairwise comparisons showed that athletes spent more time performing right than left-handed movements (*p* = 0.065).

**Table 2:** Statistical results of the within subjects effects.

Factor (multi/univariate)	F	df	*p*	Partial η^2^	Pairwise comparison
Activation					
Hand (F)	7.607	1, 18	< 0.05	0.297	rh > lh
Hand (PoT)	3.877	1, 18	= 0.065	0.177	rh > lh (*p* = 0.065)
Structure					
Hand (F)	4.953	4, 15	< 0.05	0.569	rh > lh
Hand (*phasic*) (F)	12.668	1, 18	< 0.01	0.413	
Hand (*irregular*) (F)	4.031	1, 18	= 0.060	0.183	lh > rh (*p* = 0.06)
Hand (PoT)	4.887	4, 15	< 0.05	0.566	
Hand (*phasic*) (PoT)	6.807	1, 18	< 0.05	0.274	*phasic*, rh > lh
Hand (*irregular*) (PoT)	4.878	1, 18	< 0.05	0.213	*irregular*, lh > rh
Focus					
Hand (F)	18.687	5, 14	< 0.001	0.870	*within body,* lh > rh
Hand (*within body*) (F)	7.633	1, 18	< 0.05	0.298	
Hand (*on body*) (F)	18.895	1, 18	< 0.001	0.512	*on body,* lh > rh
Hand (*on attached object*) (F)	27.423	1, 18	< 0.001	0.604	*on attached object,* lh > rh
Hand (*on separate object*) (F)	56.176	1, 18	< 0.001	0.757	*on separate object,* rh > lh
Hand * emotion (*on attached object*) (F)	6.152	1, 18	< 0.05	0.255	*on attached object,* losing > winning
Hand (PoT)	16.275	4, 14	< 0.001	0.853	*within body,* lh > rh
Hand (*within body*) (PoT)	6.099	1, 18	< 0.05	0.253	
Hand (*on body*) (PoT)	17.610	1, 18	< 0.001	0.495	*on body,* lh > rh
Hand (*on attached object*) (PoT)	26.552	1, 18	< 0.001	0.596	*on attached object,* lh > rh
Hand (*on separate object*) (PoT)	33.922	1, 18	< 0.001	0.653	*on separate object,* rh > lh
Contact					
Emotion (*PoT*)	2.580	3, 16	= 0.09	0.345	
Emotion (*act as a unit*) (*PoT*)	5.038	1, 18	< 0.05	0.219	*act as a unit*, winning > losing
Formal Relation					
Emotion (*F*)	3.056	4, 15	= 0.05	0.449	
Emotion (*left hand dominance*) (*F*)	6.782	1, 18	< 0.05	0.274	*left hand dominance*, winning > losing
Emotion (*right hand dominance*) (*F*)	3.835	1, 18	= 0.066	0.176	*right hand dominance*, winning > losing
Emotion (*PoT*)	6.427	4, 15	< 0.01	0.632	
Emotion (*left hand dominance*) (*PoT*)	11.225	1, 18	< 0.01	0.384	*left hand dominance*, winning > losing
Function					
Hand (*object-oriented action*) (F)	15.760	2, 17	< 0.001	0.467	*object-oriented action*, bh > lh *object-oriented action*, rh > lh
Hand (*subject-oriented action*) (F)	17.875	2, 17	< 0.05	0.498	*subject-oriented action*, lh > bh *subject-oriented action*, lh > bh
Emotion (F)	2.897	8, 11	< 0.05	0.685	
Emotion (*emblem/social convention*) (F)	7.080	1, 18	< 0.05	0.282	*emblem/social convention,* winning > losing
Emotion (*emotion/attitude*) (F)	12.102	1, 18	< 0.05	0.402	*emotion/attitude,* losing > winning
Emotion (*subject-oriented action*) (F)	4.542	1, 18	< 0.05	0.201	*subject-oriented action,* winning > losing
Hand (PoT)	3.353	18,58	< 0.001	0.502	
Hand (*object-oriented action*) (PoT)	15.303	2, 17	< 0.001	0.460	*object-oriented action*, rh > bh *object-oriented action*, rh > lh *object-oriented action*, bh > lh
Hand (*subject-oriented action*) (PoT)	16.360	2, 17	< 0.001	0.476	*subject-oriented action*, lh > bh *subject-oriented action*, lh > bh
Emotion (*emblem/social convention*) (PoT)	6.123	1, 18	< 0.05	0.254	*emblem/social convention,* winning > losing
Emotion (*emotion/attitude*) (PoT)	9.648	1, 18	< 0.01	0.349	*emotion/attitude,* losing > winning
Type					
Emotion (F)	10.284	12, 7	< 0.01	0.946	
Emotion (*fall*) (F)	2.309	1, 18	< 0.01	0.454	*fall*, losing > winning
Emotion (*fall*) (F)	4.542	1, 18	< 0.05	0.205	*palm-out*, losing > winning

F, Frequency; PoT, Proportion of Time; lh, left hand; rh, right hand; bh, both hands.

**Structure**. The rmANOVA for the frequency values showed a significant effect of the hand [*F*(4, 15) = 4.953, *p* < 0.05, η^2^ = 0.569]. The uANOVA showed a significant effect of the hand for phasic [*F*(1, 18) = 12.668, *p* < 0.01, η^2^ = 0.413] and a marginal effect for irregular [F(1, 18) = 4.031, *p* = 0.060, η^2^ = 0.183]. *Post-hoc* pairwise comparisons showed that athletes executed significantly more phasic hand movements with the right than the left hand (*p* < 0.01) and marginally more irregulars with the left than the right hand (*p* = 0.06). The rmANOVA for the PoT values showed a significant effect of the hand [*F*(4, 15) = 4.887, *p* < 0.05, η^2^ = 0.566]. The uANOVA showed a significant effect of the hand for phasic [*F*(1, 18) = 6.807, *p* < 0.05, η^2^ = 0.274] and irregular [F(1, 18) = 4.878, *p* < 0.05, η^2^ = 0.213]. *Post-hoc* pairwise comparisons showed that athletes spent significantly more time with phasic hand movements with the right than the left hand (*p* < 0.05) and irregular hand movements with the left than the right hand (*p* < 0.05).

**Focus**. The rmANOVA for the frequency values showed a significant effect of the hand [*F*(5, 14) = 18.687, *p* < 0.001, η^2^ = 0.870]. The uANOVA showed a significant effect of the hand for within body [*F*(1, 18) = 7.633, *p* < 0.05, η^2^ = 0.298], on body [F(1, 18) = 18.895, *p* < 0.001, η^2^ = 0.512], on attached object [F(1, 18) = 27.423, *p* < 0.001, η^2^ = 0.604], and on separate object [F(1, 18) = 56.176, *p* < 0.001, η^2^ = 0.757]. *Post-hoc* pairwise comparisons showed that the movements within body (*p* < 0.05), on body (*p* < 0.001), and on attached object (*p* < 0.001) were executed significantly more with the left hand than with the right hand. On separate object hand movements were significantly more executed with the right hand than with the left hand (*p* < 0.001). The uANOVA also showed a significant effect of the interaction of hand and emotion for on attached object [F(1, 18) = 6.152, *p* < 0.05, η^2^ = 0.255]. *Post-hoc* pairwise comparisons showed that the execution of right hand on attached object Focuses are significantly more frequent after losing than winning (*p* < 0.05).

The rmANOVA for the PoT values showed a significant effect of the hand [F(5, 14) = 16.275, *p* < 0.001, η^2^ = 0.853]. The uANOVA showed a significant effect of the hand for within body [*F*(1, 18) = 6.099, *p* < 0.05, η^2^ = 0.253], on body [F(1, 18) = 17.610, *p* < 0.001, η^2^ = 0.495], on attached object [F(1, 18) = 26.552, *p* < 0.001, η^2^ = 0.596], and on separate object [F(1, 18) = 33.922, *p* < 0.001, η^2^ = 0.653]. *Post-hoc* pairwise comparisons showed that athletes spent significantly more time using the left hand during within body (*p* < 0.05), on body (*p* < 0.001), and on attached object (*p* < 0.001) hand movements compared to the right hand. For on separate object hand movements, athletes spent significantly more time with the right hand than with the left hand (*p* < 0.001).

**Contact**. The rmANOVA for the PoT values showed no effect of emotion [*F*(3, 16) = 2.580, *p* = 0.09, η^2^ = 0.326]. The uANOVA showed a significant effect of emotion for act as a unit [F(1, 18) = 5.038, *p* < 0.05, η^2^ = 0.219]. *Post-hoc* pairwise comparisons showed that athletes spent significantly more time with act-as-a-unit hand movements after winning than after losing (*p* < 0.05).

**Formal Relation**. The rmANOVA for the frequency values showed a marginal effect of emotion [*F*(4, 15) = 3.056, *p* = 0.05, η2 = 0.449]. The uANOVA showed a significant effect of emotion for left-hand dominance [*F*(1, 18) = 6.782, *p* < 0.05, η^2^ = 0.274], and a marginal effect for right-hand dominance [F(1, 18) = 3.835, *p* = 0.066, η^2^ = 0.176]. *Post-hoc* pairwise comparisons showed that athletes executed significantly more left-hand dominance (p < 0.05) movements and marginally more right-hand dominance (*p* = 0.066) movements after winning than after losing.

The rmANOVA for the PoT values showed a significant effect of emotion [F(4, 15) = 6.427, *p* < 0.01, η^2^ = 0.632]. The uANOVA showed a significant effect of emotion for left-hand dominance [*F*(1, 18) = 11.225, *p* < 0.01, η^2^ = 0.384]. *Post-hoc* pairwise comparisons showed that athletes spent significantly more time with left-hand dominance movements after winning than after losing (*p* < 0.01).

**Function**. The uANOVA for the frequency values showed a significant effect of hand for object-oriented action [*F*(2, 17) = 15.760, *p* < 0.001, η^2^ = 0.467] and subject-oriented action [F(2, 17) = 17.875, *p* < 0.001, η^2^ = 0.498]. *Post-hoc* pairwise comparisons showed that athletes executed significantly more object-oriented actions with both hands (*p* < 0.001) and with the right hand (*p* < 0.001) than with the left hand. Subject-oriented actions were executed significantly more often with the left hand compared to both hands (*p* < 0.001) and to right hand executions (*p* < 0.01).

**Figure 2:**
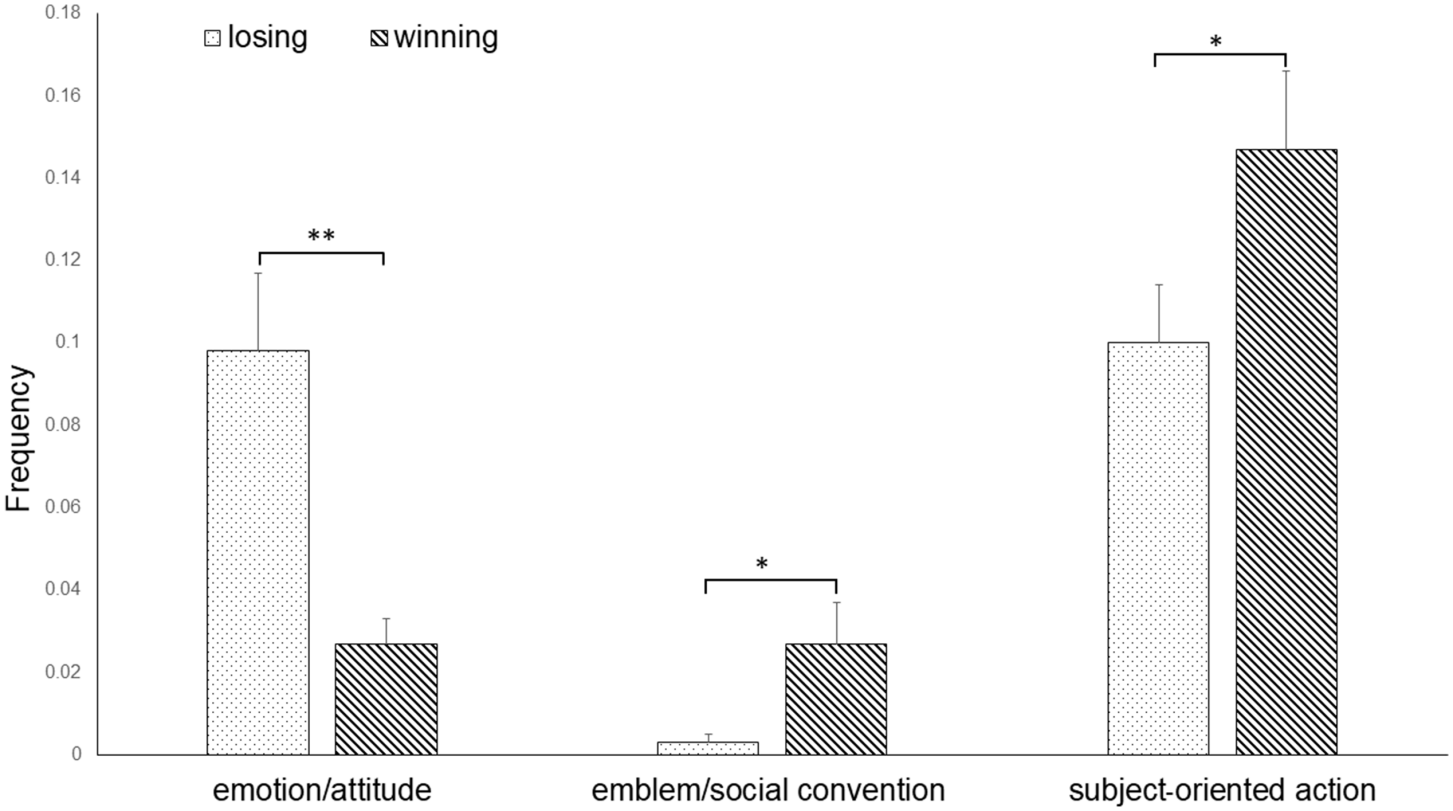
Emotion/attitude and emblem/social convention gestures as well as subject-oriented actions (Function) of athletes when winning or losing during a competition.

The rmANOVA showed a significant effect of emotion [*F*(8, 11) = 2.897, *p* < 0.05, η^2^ = 0.685]. The uANOVA showed a significant effect of emotion for emblem/social convention [*F*(1, 18) 7.080, *p* < 0.05, η^2^ = 0.282], emotion/attitude [F(1, 18) = 12.102, *p* < 0.05, η^2^ = 0.402], and subject-oriented action [F(1, 18) = 4.542, *p* < 0.05, η^2^ = 0.201]. *Post-hoc* pairwise comparisons showed that athletes executed significantly more emblem/social convention (*p* < 0.05) gestures and subject-oriented actions (*p* < 0.05) after winning than losing. Emotion/attitude gestures were significantly increased after losing compared to winning (*p* < 0.01; Figure 2).

The rmANOVA for the PoT values showed a significant effect of hand [*F*(18, 58) = 3.253, *p* < 0.001, η^2^ = 0.502].The uANOVA showed a significant effect of hand for object-oriented action [*F*(2, 36) = 15.303, *p* < 0.001, η^2^ = 0.460] and subject-oriented action [F(2, 36) = 16.360, *p* < 0.001, η^2^ = 0.476]. *Post-hoc* pairwise comparisons showed that athletes spent significantly more time with object-oriented actions with the right hand than with both hands (*p* < 0.05) or the left hand (*p* < 0.01) and significantly more time with both hands than with the left hand (*p* < 0.001). They spent significantly more time with subject-oriented actions with the left hand compared to both hands (*p* < 0.001) and to the right hand (*p* < 0.01).

The uANOVA showed a significant effect of emotion for emblem/social convention [*F*(1, 18) = 6.123, *p* < 0.05, η^2^ = 0.254] and emotion/attitude [F(1, 18) = 9.648, *p* < 0.01, η^2^ = 0.349]. *Post-hoc* pairwise comparisons showed that athletes spent significantly more time performing emblem/social convention gestures after winning than losing (*p* < 0.05). Athletes also spent significantly more time performing emotion/attitude hand movements after losing than winning (*p* < 0.01).

**Figure 3:**
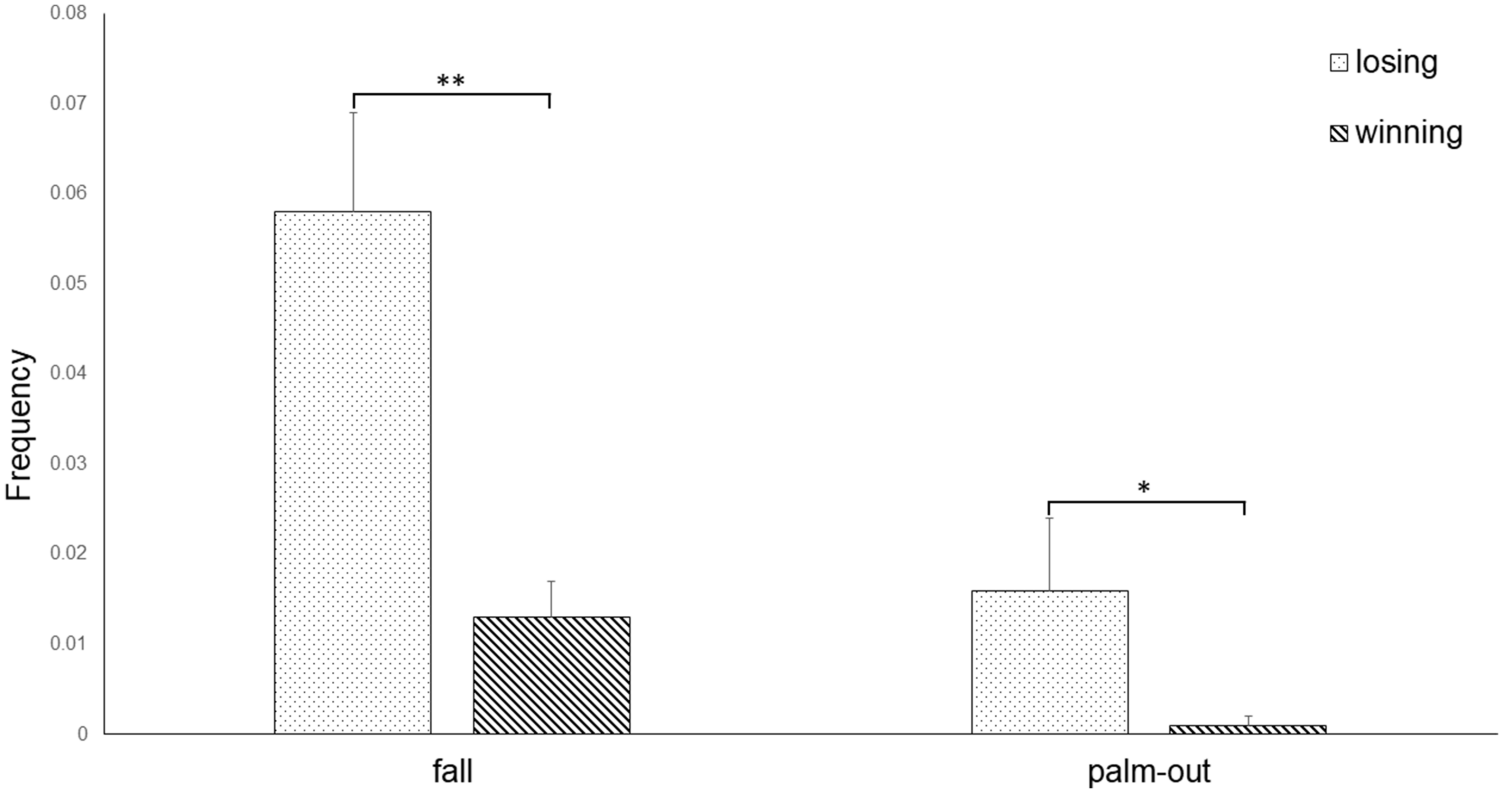
Fall and palm-out gestures (Type) of athletes when winning or losing during competition.

**Type**. The rmANOVA for the frequency values showed a significant effect of emotion [*F*(12, 7) = 10.284, *p* < 0.01, η^2^ = 0.946]. The uANOVA showed a significant effect of emotion for fall [*F*(1, 18) = 2.309, *p* < 0.01, η^2^ = 0.454] and palm-out [F(1, 18) = 4.542, *p* < 0.05, η^2^ = 0.205]. *Post-hoc* pairwise comparisons showed that athletes executed significantly more fall (*p* < 0.01) and palm-out (*p* < 0.05) gestures after losing than winning (Figure 3).

### 3.2 Research question 2

*How do nonverbal behaviors differ between female and male athletes during positive and negative affect situations?*

**Activation**. No significant effects were found between groups.

**Figure 4:**
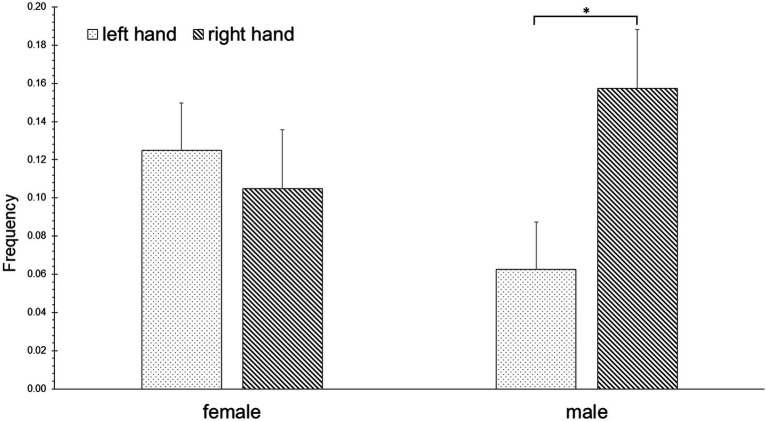
Repetitive hand movements (Structure) of female and male athletes with the right and left hand.

**Structure**. The rmANOVA of the frequency values showed a marginally significant effect of the interaction of group and hand [*F*(4, 15) = 2.694, *p* = 0.071, η^2^ = 0.418; Table 3]. The uANOVA showed a significant effect of the interaction of group and hand for repetitive movements [F(1, 18) = 4.677, *p* < 0.05, η^2^ = 0.206]. *Post-hoc* pairwise comparisons showed that male athletes executed significantly more repetitive hand movements with the right hand than with the left hand (*p* < 0.05; Figure 4).

**Table 3:** Statistical results of gender (between subjects) effects.

Factor (multi/univariate)	F	df	*p*	Partial η^2^	Pairwise comparison
Activation					
No significant effects					
Structure					
Group * hand (F)	2.694	4, 15	= 0.071	0.418	male, *repetitive*, rh > lh
Group * hand (*repetitive*) (F)	4.677	1, 18	< 0.05	0.206	
Group * hand (PoT)	3.088	4, 15	< 0.05	0.418	male, *repetitive*, rh > lh female, *phasic*, rh > lh
					female, *aborted*, rh > lh
Focus					
Group (PoT)	4.206	5, 14	< 0.05	0.600	female, *in space* > male
Group (*in space*) (PoT)	9.484	1, 18	< 0.01	0.345	
Group * hand (*in space*) (PoT)	6.314	1, 18	< 0.05	0.260	female, rh, *in space* > male female, rh, *in space* > lh male, female, *on attached object*, lh > rh
Group * hand (*on attached object*) (PoT)	4.507	1, 18	< 0.05	0.200	
Contact					
Group (*act as a unit*) (F)	8.757	1, 18	< 0.01	0.327	female, *act as a unit* > male
Formal Relation					
No significant effects					
Function					
Group * hand * emotion (*pantomime*) (F)	4.281	2, 17	< 0.05	0.192	female, bh, losing, *pantomime* > male (p = 0.074)
Group * hand * emotion (*subject-oriented action*) (F)	2.947	2, 17	= 0.065	0.141	male, bh, winning, *subject-oriented action* > female; female, lh, losing, *subject-oriented action* > rh; male, lh, losing, *subject-oriented action* > rh
Group * hand * emotion (*pantomime*) (PoT)	4.333	2, 17	< 0.05	0.194	female, bh, losing, *pantomime* > winning
Type					
Group (*fall*) (F)	4.175	1, 18	= 0.056	0.188	female, *fall* > male (*p* = 0.056)
Group (*fall*) (PoT)	4.295	1, 18	= 0.03	0.193	female, *fall* > male (*p* = 0.053)

F, Frequency; PoT, Proportion of Time; lh, left hand; rh, right hand; bh, both hands.

The rmANOVA of the POT values showed a significant effect of the interaction of group and hand [F(4, 15) = 3.088, *p* < 0.05, η^2^ = 0.452]. The uANOVA did not show significant effects. *Post-hoc* pairwise comparisons showed that female athletes spent significantly more time with phasic movements with the right hand when compared to the left hand (*p* < 0.01), as well as more time with aborted movements with the left hand when compared to the right hand (*p* < 0.05). Male athletes spent more time in repetitive movements with the right hand compared to the left hand (*p* < 0.05).

**Focus**. Neither the rmANOVA nor the uANOVA showed significant results for the frequency values. The rmANOVA for the PoT values showed a significant effect for group [*F*(5, 14) = 4.206, p < 0.05, η^2^ = 0.600]. Furthermore, the rmANOVA showed significance for in-space movements [*F*(1, 18) = 9.484, *p* < 0.01, η^2^ = 0.345]. *Post-hoc* pairwise comparisons showed that female athletes spent significantly more time performing in-space movements than male athletes (*p* < 0.01).

Furthermore, the uANOVA showed a significant interaction effect of hand and group for in-space [F(1, 18) = 6.314, *p* < 0.05, η^2^ = 0.260] and on attached object movements [F(1, 18) = 4.507, *p* < 0.05, η^2^ = 0.200]. *Post-hoc* pairwise comparisons showed that female athletes spent significantly more time performing in-space hand movements with the right hand than male athletes (*p* < 0.001). Further, *post-hoc* pairwise comparisons showed that female athletes (but not male athletes) spent significantly more time performing in-space movements with the right hand compared to the left hand (*p* < 0.05). For on attached object movements, male (*p* < 0.001) and female athletes (*p* < 0.05) both spent more time using their left hand than right hand.

**Contact**. The rmANOVA for the frequency values showed a significant group effect for act-as-a-unit movements [F(1, 18) = 8.757, *p* < 0.01, η^2^ = 0.327]. *Post-hoc* pairwise comparisons showed that female athletes executed significantly more act-as-a-unit hand movements than male athletes (*p* < 0.01).

**Formal Relation**. No significant effects were found between groups.

**Function**. The uANOVA for the frequency values showed a significant effect of the interaction of hand, emotion, and group for pantomime [*F*(2, 17) = 4.281, *p* < 0.05, η^2^ = 0.192] and a marginal effect for subject-oriented actions [F(2, 17) = 2.947, *p* = 0.065, η^2^ = 0.141]. *Post-hoc* pairwise comparisons showed that female athletes executed marginally more pantomime gestures with both hands after losing than winning (*p* = 0.074). Male athletes executed more subject-oriented actions with both hands when winning than women (*p* < 0.05). Men further showed significantly more subject-oriented actions with both hands after winning compared to losing (*p* < 0.01). Furthermore, after winning, women showed significantly more left-hand subject-oriented actions than right hand (*p* < 0.01) and both hands (*p* < 0.01). After losing, women showed significantly more left-hand subject-oriented actions than right hand (*p* < 0.05) and both hands (*p* < 0.05). Men also showed significantly more left-hand subject-oriented actions than both hands when losing (*p* < 0.05).

**Figure 5:**
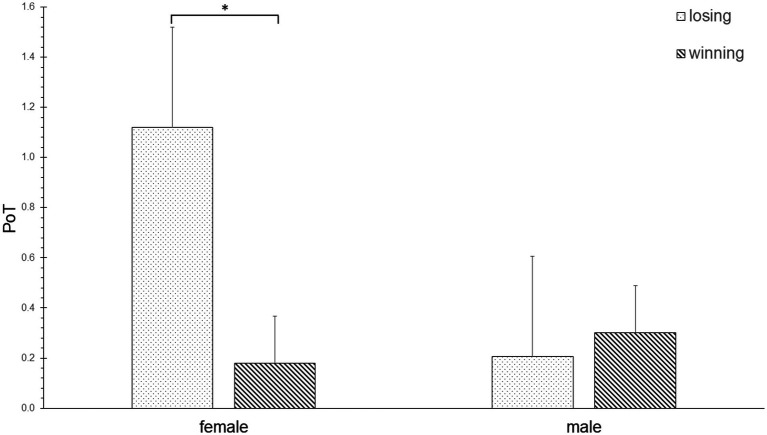
Pantomime gestures (Function) of female and male athletes.

The uANOVA for the PoT values showed a significant effect of the interaction of hand, emotion, and group for pantomime [F(2, 17) = 4.333, *p* < 0.05, η^2^ = 0.194]. *Post-hoc* pairwise comparisons showed that female (not male) athletes spent significantly more time performing pantomime gestures with both hands after losing when compared to winning (*p* < 0.05, Figure 5).

**Type**. The rmANOVA for the frequency values showed a marginal effect between groups for fall [F(1, 18) = 4.175, *p* = 0.056, η^2^ = 0.188]. *Post-hoc* pairwise comparisons showed that female athletes executed marginally more fall gestures than male athletes (*p* = 0.056).

The rmANOVA for the PoT values showed a marginal effect between groups for fall [F(1, 18) = 4.295, *p* = 0.053, η^2^ = 0.193]. *Post-hoc* pairwise comparisons showed that female athletes spent marginally more time performing fall gestures than men (*p* = 0.053).

## 4 Discussion

The aim of the present study was twofold. The first aim of the study was to investigate how nonverbal behaviors differ during positive and negative affect in semi-professional athletes. More specifically, we tested the hypothesis that positive affective states (after winning points in tennis) lead to lateralized body-distant movements and negative affective states (after losing a point) lead to on-body movements in an attempt to regulate these negative affective states. The second aim was to explore how nonverbal behaviors differ between female and male athletes during positive and negative affect situations.

Regarding the first research question, all athletes executed more right-handed movements overall. However, for phasic and on separate object hand movements, as well as object-oriented actions, athletes showed a right-hand preference. Irregular, within body, on body, on attached object, and subject-oriented actions were mostly executed with the left hand. The effect of positive and negative affect was observed by increased executions (mostly with the left hand) of on attached object hand movements as well as emotion/attitude, fall, and palm-out gestures after losing points. In contrast, athletes executed more act-as-a-unit hand movements, more left- and right-hand dominance, and more emblem/social convention gestures and subject-oriented actions after winning than to losing.

Regarding the second research question, the comparison of the entire nonverbal hand movement and gestural behavior of female and male athletes during positive and negative affect situations in sports (i.e., when winning or losing points) revealed that the two genders are not characterized by greater or fewer nonverbal hand movements overall. However, the two genders express distinct nonverbal hand movements and gestures that serve different neuropsychological functions. Female athletes showed more phasic, in space, and act-as-a-unit hand movements as well as pantomime and fall gestures than male athletes. Female but not male athletes executed more pantomime gestures with both hands after losing than winning. Male athletes expressed more repetitive hand movements with the right hand and more subject-oriented actions with both hands when winning and when compared to female athletes.

### 4.1 Research question 1

Independently from group effects, this study found alterations in the nonverbal behavior of athletes in response to the (positive / negative) affect situations as well in the right and / or the left hand (and / or both hands) for several categories of the NEUROGES. Overall, the present data showed that athletes execute more nonverbal hand movements with the right hand. Although this may not be surprising as the study integrated right-handed athletes only, it is still significant as right-handed athletes would hold their tennis racket with the right hand. However, Neumann et al. (2021) already showed that professional tennis athletes take their racket with their left hand in order to express emotional gestures with their right hand. When analyzing the Structure category, the present data showed that female and male athletes would execute phasic hand movements with a preference to the right hand but irregular hand movements with a left-hand preference. Such lateralities have been observed previously in studies that applied the NEUROGES system in several populations (Helmich et al., 2014, 2021; Helmich and Lausberg, 2019; Neumann et al., 2021). Because phasic hand movements are characterized by a phase structure and a complex motion phase (Lausberg, 2013), these hand movements are considered cognitively more complex and may rely on left hemispheric motor-cognitive processes (Helmich et al., 2021; Helmich and Lausberg, 2014; Neumann et al., 2021). The lateralization to the left hand of irregular hand movements also supports previous studies (Helmich and Lausberg, 2014, 2019; Neumann et al., 2021). Irregular movements are colloquially known as fidgeting and mostly act on the body. In the present study, on-body hand movement Focuses showed to be lateralized to the left hand as well. A left-hand preference has been commonly observed for self-touching (Saucier and Elias, 2001; Sousa-Poza et al., 1979; Trevarthen, 1996). Thus, this study replicates previous findings of nonverbal hand lateralization (Helmich et al., 2021, 2024; Helmich and Lausberg, 2014; Helmich and Schepmann, 2023; Neumann et al., 2021). Irregular on-body hand movements have been commonly observed during negative emotions, in symptomatic patients with post-concussion symptoms, and / or in depressive patients (Barroso et al., 1978; Densing et al., 2018; Helmich and Lausberg, 2019; Reinecke et al., 2021; Ulrich and Harms, 1985). In fact, previous studies on professional athletes showed that irregular on-body movements are increased in response to losing points in tennis (Neumann et al., 2021). In the present study, we did not find significantly increased irregular on-body movements in response to losing points. Differences may be grounded in the fact that Neumann et al. (2021) investigated professional tennis athletes during public matches (with spectators). Assuming that irregular on-body hand movements particularly increase in emotional and / or stressful situations, the atmosphere during matches in the 4th tennis league of Germany (as in the present study) may be less intense as there were not as many spectators as during first league matches. Thus, athletes may nonverbally self-regulate more intensively during events with more spectators. However, at this point the hypothesis of whether more spectators increase irregular on-body hand movements in athletes must be clarified in further studies.

Still, the present data further showed that female and male athletes spontaneously increase their on-attached-object (e.g., the t-shirt) hand movements when losing compared than winning points. On-attached-object hand movements were also lateralized to the left hand. Thus, athletes nonverbally focus on themselves when experiencing negative emotions. It has been observed that self-touches alter brain functions in a way that indicates regulation of attentional, emotional, and working memory processes (Spille et al., 2022). Thus, here the athletes show a different kind of self-touch (on attached object) that may serve similar functions as to previously observed irregular on-body hand movements (Neumann et al., 2021) and / or by touching the face (Spille et al., 2022). Still, Neumann et al. (2021) observed increased on-attached-object hand movements in professional athletes when losing compared to when winning. Thus, it seems evident that professional as well as semi-professional athletes spontaneously increase their nonverbal movement behavior toward their own body that indicates self-regulative functions when losing points in tennis.

Furthermore, both genders not only showed particular hand movements in relation to winning and losing points in tennis but also showed different gestures (Function and Type codings of the NEUROGES). Emblem/social convention gestures were increased when winning. The latter gestures are defined as “using culture-specific hand signs with conventionalized arbitrary meanings / conventionalized actions in specific social contexts” (Lausberg, 2013). Here, tennis athletes used such gestures when winning. Those gestures were mostly pointing the index finger in an upwards direction, e.g., when to nonverbally signal to the opponent that a ball was out. Those gestures were not observed in a previous study with a similar design but using professional athletes (Neumann et al., 2021). The difference may be grounded in the fact that Neumann et al. (2021) investigated professional athletes playing in the first league (/1st Bundesliga) of Germany. During professional tournaments a referee is present during matches (Deutscher Tennis Bund, 2021). In the present study, semi-professional tennis athletes played without referees. Thus, they must inform the opponent if a ball is in or out. To indicate this, the athletes executed more emblem/social convention gestures to inform the opponent about errors (e.g., pointing the left index finger toward the sky to indicate “the ball was out”). The latter gestures are therefore not strongly related to emotional expressions but rather serve communication and/or signaling purposes. In fact, the athletes in this study did not show as many specific wining gestures such as emotional rise gestures (“Becker-Faust”) that were observed in professional athletes (Neumann et al., 2021). Here, winning points resulted in more subject-oriented actions, i.e., hand movements that are focused on the body to change the physical (and secondarily mental) state (Lausberg, 2013). The difference may also be related to the setting, i.e., professional versus semi-professional and the presence/absence of spectators. This may have resulted in more expressive winning gestures such as the so-called “Becker-Faust” gesture (Neumann et al., 2021). Here, athletes instead focused on their own body through subject-oriented actions. It has been previously shown that self-touches increase attentional processes (Barroso et al., 1978; Spille et al., 2022). We therefore conclude that semi-professional female and male athletes nonverbally focus on their own body through more subject-oriented actions in order to keep performing during tennis matches.

### 4.2 Research question 2

The present study showed that female and male athletes do not behave fundamentally differently in their nonverbal movement behavior overall but show gender-specific hand movements and gestures in response to winning and losing points in tennis. The Activation category considers any hand movement that is executed independently from its Structure, Focus, Function, etc. It therefore provides a “general impression about an individual’s level of motor activity” (Lausberg, 2013). Both genders move equally frequently and in time overall. However, the analyses of further categories of the NEUROGES system showed that the two genders are characterized by distinct nonverbal hand movements and gestures during emotional situations in tennis. Female athletes showed more phasic and in-space hand movements, particularly with the right hand. Phasic hand movements constitute movements with a phase structure such as a preparation, stroke, and retraction. Thus, phasic hand movements are conceptualized and complex (Lausberg, 2013). Phasic hand movements are often executed in space, i.e., acting without touching something. Female athletes also executed more phasic Structures with the right hand, while male athletes executed more repetitive hand movement Structures. The only difference between the latter structures constitutes the stroke phase, which is characterized by a single (/phasic) or a repetitive movement path (Lausberg, 2013). Because male athletes behave repetitively but females execute more phasic hand movements, i.e., execute more conceptualized hand movements with a single stroke, female athletes may be conceptually more controlled than male athletes during emotional situations in sports. Bjorklund and Kipp (1996) postulated that there is a female advantage in inhibition and self-regulation (Bjorklund and Kipp, 1996). Thus, emotional situations result in different hand movements (Structure and Focus of the NEUROGES) in male and female athletes, demonstrated by the fact that female athletes express more explicit hand movements than men.

This observation gains further strength as the data also showed that women expressed more act-as-a-unit hand movements, i.e., hand movements with both hands touching with a fixed configuration and taking a joint action (Lausberg, 2013). The Functional analysis (of the NEUROGES) showed that women expressed more pantomime gestures that were mostly expressed with both hands when losing points. Thus, it seems that female tennis athletes express emotional states nonverbally in response to losing points not only through more conceptualized hand movements (such as phasic) but specifically by performing more pantomime gestures with both hands. Here, pantomime gestures with both hands mostly constituted a movement such as a simulation of “how to hit the ball.” Thus, women may have simulated a “corrected version” of their tennis stroke nonverbally as a consequence of losing a point. This indicates that women explicitly express a plan to solve the next action rather than to implicitly respond to the previous point that was lost.

It has been previously shown that men may expend less effort when using cognitive regulation, perhaps due to greater use of automatic emotion regulation. Women may use positive emotions in the service of reappraising negative emotions to a greater degree (McRae et al., 2008). Women also showed more sustained performance during test-taking than males (Balart and Oosterveen, 2019). Thus, women seem to nonverbally behave more strategically during emotional situations than men in order to plan the next action. We conclude that women act nonverbally in a more explicit and therefore strategic way to self-regulate emotional situations in sports in order to control performance.

A further observation has been made within the Type category through the increased frequencies of fall gestures in women than male athletes. Fall gestures constitute a subcategory of emotion/attitude gestures. Female athletes let their arms fall down heavily when losing a point in tennis significantly more frequently than male athletes. This indicates that, first, implicit gestures after losing points in tennis increase more during negative emotional states and are accompanied by fall gestures; secondly, this negative emotional gesture appears more often in women than men. Negative emotional periods or depression are more prevalent in women (Albert, 2015). The hypothesis explaining why women may experience depression more frequently concerns constitute excessive empathy, compliance, and regulation of negative emotions (Keenan and Hipwell, 2005). Nonverbal emotional hand movements related to negative thoughts and arousal have been previously formulated (Barroso et al., 1978; Ulrich and Harms, 1985). Thus, the present data about increased fall gestures related to negative affect in women may represent the fact that women tend to be nonverbally more expressive, particularly when negative thoughts are being processed. Together with the fact that female athletes present more conceptualized (phasic), in-space hand movements and pantomime gestures (with both hands acting as a unit) when losing points indicates that woman are able to transfer their negative thoughts into conceptualized movements to improve future actions. Thus, female athletes express emotions nonverbally more than male athletes do but also exhibit better control over such emotions using nonverbal strategies. In fact, women have been shown to use more emotional regulation strategies than men and be more flexible in the implementation of those strategies (Goubet and Chrysikou, 2019). Thus, we conclude that women increase spontaneous emotional expressions (fall gestures) during sports but are also characterized by increased hand movements and gestures that serve to explicitly regulate their negative emotional arousal.

## 5 Conclusion

This study recorded and analyzed for the first time all the nonverbal hand movement and gestural behavior of female and male athletes during competitive tennis matches. Particular hand movements and gestures that serve particular neuropsychological functions characterize female and male athletes during emotional situations in sports. The fact that female athletes express more conceptualized/controlled hand movements (e.g., pantomime gestures with both hands) than men do indicates that women not only express more emotions nonverbally but also that they act more strategically during emotional situations in sports. We assume that, despite experiencing negative emotions, female athletes explicitly plan their next actions which is observable through their nonverbal behavior. Thus, female athletes better cope with their negative emotional arousal using explicit hand movements and gestures to control future performances.

## Data Availability

The raw data supporting the conclusions of this article will be made available by the authors, without undue reservation.
